# Fluid-Structure Interaction Analysis on Membrane Behavior of a Microfluidic Passive Valve

**DOI:** 10.3390/membranes10100300

**Published:** 2020-10-21

**Authors:** Zhen-hao Lin, Xiao-juan Li, Zhi-jiang Jin, Jin-yuan Qian

**Affiliations:** 1 Institute of Process Equipment, College of Energy Engineering, Zhejiang University, Hangzhou 310027, China; linzhenhao@zju.edu.cn (Z.-h.L.); lixiaojuan@zju.edu.cn (X.-j.L.); jzj@zju.edu.cn (Z.-j.J); 2 State Key Laboratory of Fluid Power and Mechatronic Systems, Zhejiang University, Hangzhou 310027, China

**Keywords:** microfluidic passive valve (MPV), fluid-structure interaction (FSI), flow rate, threshold pressure, elastic membrane

## Abstract

In this paper, the effect of membrane features on flow characteristics in the microfluidic passive valve (MPV) and the membrane behavior against fluid flow are studied using the fluid-structure interaction (FSI) analysis. Firstly, the microvalve model with different numbers of microholes and pitches of microholes are designed to investigate the flow rate of the MPV. The result shows that the number of microholes on the membrane has a significant impact on the flow rate of the MPV, while the pitch of microholes has little effect on it. The constant flow rate maintained by the microvalve (the number of microholes *n* = 4) is 5.75 mL/min, and the threshold pressure to achieve the flow rate is 4 kPa. Secondly, the behavior of the membrane against the fluid flow is analyzed. The result shows that as the inlet pressure increases, the flow resistance of the MPV increases rapidly, and the deformation of the membrane gradually becomes stable. Finally, the effect of the membrane material on the flow rate and the deformation of the membrane are studied. The result shows that changes in the material properties of the membrane cause a decrease in the amount of deformation in all stages the all positions of the membrane. This work may provide valuable guidance for the optimization of microfluidic passive valve in microfluidic system.

## 1. Introduction

Microfluidics, as an emerging technology, deals with small volumes of fluids utilizing the microchannel with tens to hundreds of microns in size [1]. It has the advantages of low sample consumption and energy consumption, low cost, high security, etc., which is widely used in many fields, including chemistry [2,3], biochemistry [4,5], biomedicine [6], biotechnology [7], etc. In order to achieve fluid control and operation at a micro-scale, several main components, including microsensors, micropumps, microvalves, micromixers, and microchannels, comprise a microfluidic system [8]. Among those components, a compact and efficient microvalve is important to control the flow accurately. The precise manipulation of microfluidics is a key part of sample processing and analysis, which directly determines the reliability of the microfluidic system.

The microvalve, depending on their structure, can be divided into two types: active microvalve and passive microvalve [9]. Comparing with the active microvalve, utilization of passive microvalve avoids the introduction of external components for flow control. Thus, it is simpler, more reliable, and less expensive. Elastic membranes are adopted to realize the functions of flow regulation or on/off switching in the passive microvalve. Many microvalves with an elastic membrane have been reported in previous research. In a passive microvalve operating condition, internal fluid pressure drives the elastic membrane to deform, so as to control the flow rate or on/off switching. This technology, which used elastic membranes to control flow, has been widely applied in the passive microvalve of the microfluidic systems [8,9,10]. The main membrane materials that have been reported are shape memory alloy [11], thermal plastic polymer [12,13], glass [14], and polydimethylsiloxane (PDMS). Among those membrane materials, the PDMS has excellent optical transparency and higher elasticity, gas permeability, and biocompatibility [15,16]. Thus, it is widely adopted to the throttle element of passive microvalves. Passive microvalve with PDMS membrane further improves the integration of the microfluidic system. When the microvalve is working condition, the deformation of the membrane is closely related to the flow characteristics in the microvalve. Computational fluid dynamics and experimental methods are often adopted to analyze flow characteristics in valves [17,18]. Moreover, some researchers performed experimental or FSI analysis to investigate the flow characteristics and membrane behavior against fluid flow of the microvalves. Kartalov et al. [19] fabricated a PDMS push-up valve, and the change of flow rate in the microvalve with different pressure is experimentally studied. The experimental results showed that the constant flow rate was 0.033 mL/min when the threshold pressure reached 103 kPa. Doh et al. [20] fabricated a parallel membrane valve, and experimentally found that the pressure threshold to reach a constant flow rate was 15 kPa. The constant flow rate of this microvalve was 0.83 mL/min. Jaemin et al. [21] performed an experimental analysis on different shaped structures in normally closed microvalve models. The study found that the microvalve can achieve a near-low constant opening threshold pressure regardless of the change in the input flow rate. Later, Natarajan et al. [22] performed the FSI analysis on two different models (taking from [21]) in normally closed microvalve models. The characteristics of fluid flow and the behavior of membrane deformation inside the normally closed microvalve were observed. Chakraborty et al. [23] fabricated a single-channel microfluidic device with PDMS elastic membrane. The membrane deformation and pressure drop of the microfluidic device were tested through experiments and FSI analysis. Zhang et al. [24] fabricated a microfluidic passive valve that can achieve the constant flow rate at a lower threshold pressure, i.e., the constant flow rate of the microvalve was 4.03 mL/min when the threshold pressure reached 6 kPa. This microvalve has a lower threshold pressure and better regulation performance of flow rate than the previously reported passive microvalves. Although many studies have focused on the flow characteristics inside microvalve in recent years, relatively fewer studies have focused on the membrane behavior against fluid flow using FSI analysis.

In this paper, a two-way FSI analysis is performed aiming at a microfluidic passive valve (MPV) using ANSYS Workbench 17.2 (Ansys, Canonsburg, PA, USA). The effect of membrane features on flow characteristics in the MPV and the membrane behavior against fluid flow are investigated. Firstly, the microvalve model with different numbers of microholes and pitches of microholes are used to investigate the flow regulation performance of the MPV. Moreover, the performance and behavior of the membrane against the fluid flow are analyzed. In addition, the effect of the membrane material on the flow regulation performance and the deformation of the membrane are studied. This work may provide valuable guidance for the optimization of microfluidic passive valve in microfluidic system.

## 2. Numerical Methods

### 2.1. Microvalve Model

**Figure 1:**
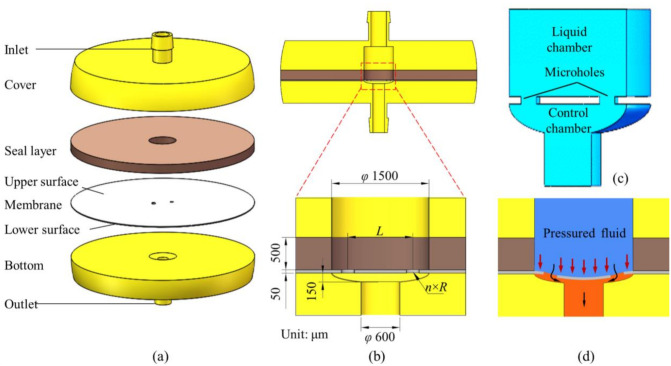
(**a**) Three-dimensional (3D) model of the MPV, (**b**) critical dimensions of the MPV, (**c**) fluid domain, and (**d**) schematic diagram of the MPV actuation under pressurized fluid.

The three-dimensional (3D) model of the MPV is shown in Figure 1. The microvalve structure consists of a cover, seal layer, membrane, and bottom. The internal flow channel is divided into three parts: a liquid chamber, microholes and control chamber, as shown in Figure 1c. The flow regulation performance of the microvalve is achieved by the deformation of the membrane under the action of the fluid pressure, as shown in Figure 1d. Zhang et al. [24] have verified the flow regulation performance of the microfluidic passive valve experimentally.

**Figure 2:**
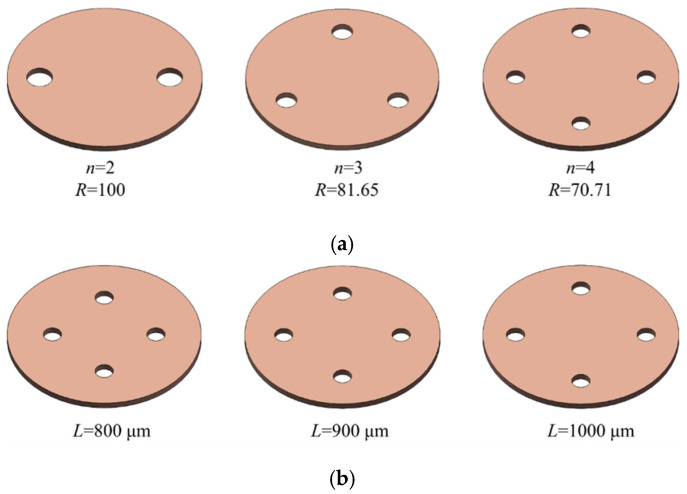
The microholes design on the PDMS membrane. (**a**) Number of microholes (*L* = 1000 μm), (**b**) different pitches of microholes (*n* = 4).

The parameters of the MPV are consistent with Zhang et al. [24]. The diameter of the liquid chamber is 1500 μm. The thickness of the seal layer is 500 μm. The control chamber is set as an ellipsoid with a diameter of 1500 μm and a depth of 150 μm. The outlet diameter is 600 μm. The membrane is set to *n* microholes, which are evenly distributed around the ring, the pitch of the microhole is *L*, the diameter of the microhole is 2R, and the thickness of the membrane is 50 μm, as shown in Figure 2. Different designs of the membrane are adopted to achieve good performance of flow control. The number of microholes *n* is 2, 3, and 4 based on the principle of constant flow area in MPV. The pitch of microholes *L* is 800 μm, 900 μm, and 1000 μm. The membrane material properties of PDMS are listed in Table 1.

### 2.2. Mathematical Model in FSI

**Figure 3:**
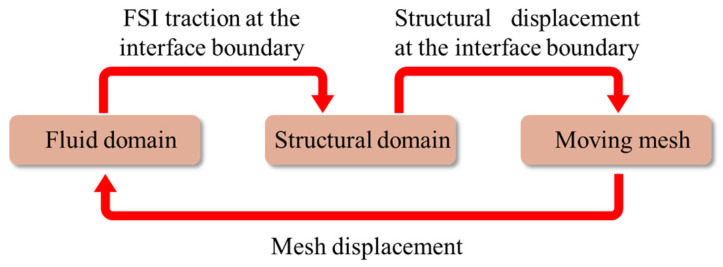
Schematic diagram of FSI.

FSI analysis of the two-way coupling method transfers the pressure calculated by fluid domain to the solid domain, and the nodal displacement calculated by the solid domain is returned to the fluid domain to update the flow field [25]. This coupling method needs to consider every field, i.e., fields that including the fluid flow, structural deformations, and moving mesh need to be considered during the coupling process, as shown in Figure 3. The FSI analysis is carried out in ANSYS Workbench 17.2. The fluid field and solid field are solved in ANSYS-FLUENT and ANSYS-Transient Structure based on the given boundary conditions, respectively. The governing equations involved in the coupling analysis are as follows [26,27].

#### 2.2.1. Fluid Formulation

The momentum conservation equation and the continuity equation of incompressible flow are as follows. Navier-Stokes equation is adopted to solve the fluid problem. It includes an arbitrary Lagrangian-Eulerian (ALE) framework:

(1) $$\rho_{f}\frac{\partial v}{\partial t}{|}_{x}+\rho \left(v-v^{m}\times |\nabla v\right)+\nabla p=\rho b^{f}+2\mu_{f}\nabla \times \nabla^{s}$$

(2) $$\nabla \times \vec{v}=0$$

where $\rho_{f}$ refers to fluid density (kg/m^3^); $\mu_{f}$ refers to the viscosity (kg/m·s); *v* refers to velocity (m/s); *p* refers to pressure (MPa); *v^m^* refers to the mesh velocity (m/s); *b^f^* refers to body force vector; $\frac{\partial v}{\partial t}{|}_{x}$ refers to the time derivative of the mesh; $\nabla^{s}v=\left(\nabla v+{\left(\nabla v\right)}^{T}/2\right)$ refers to rate-of deformation tensor.

In this paper, an incompressible, viscous fluid is applied, and the behavior of the elastic membrane is analyzed. The working fluid is water and the dimension parameters are shown in Figure 1b. The Reynolds number is calculated as:

(3) Re=ρfvDμf≈370.69<2300

where *D* refers to the hydraulic diameter. Thus, fluid flow in the MPV is considered laminar in this paper.

#### 2.2.2. Solid Formulation

In the solid continuum domain, the conservation equation of linear momentum with respect to spatial coordinate *x* is as follows. It follows a standard Lagrangian description:

(4) $$\nabla \left(FS^{s}\right)+\rho_{s}b^{s}=\frac{\partial^{2}u}{\partial t^{2}}$$

where $\rho_{s}$ refers to solid density (kg/m^3^); u refers to the displacement field (mm); *b^s^* refers to the body force vector; F=I+∇u refers to the deformation gradient tensor.

#### 2.2.3. FSI Interface Conditions

The interface data exchange formulation between solid and fluid is as follows, which include the kinematic and dynamic constraints specified:

(5) $$v^{f}=\frac{\partial u^{s}}{\partial t}$$

(6) $$\sigma^{s}\cdot n_{s}=-\sigma^{f}\cdot n_{s}$$

where *n_s_* refers to the outer normal at the solid. 

#### 2.2.4. Moving Mesh

The fluid boundary conditions come from the solid deformation when the ALE is adopted in the FSI analysis. The method based on solving the linear elasticity equation is adopted; thus, the governing equations of fluid mesh nodes are follows:

(7) $$\nabla \cdot \sigma^{p-m}=0$$

(8) $${\left(u_{i}\right)}^{p-m}={\left(u_{i}\right)}^{b}$$

where $\sigma^{p-m}$ refers to Cauchy stress tensor [26]; *u^b^* refers to the boundary displacement vector.

### 2.3. Mesh and Boundary Conditions

**Figure 4:**
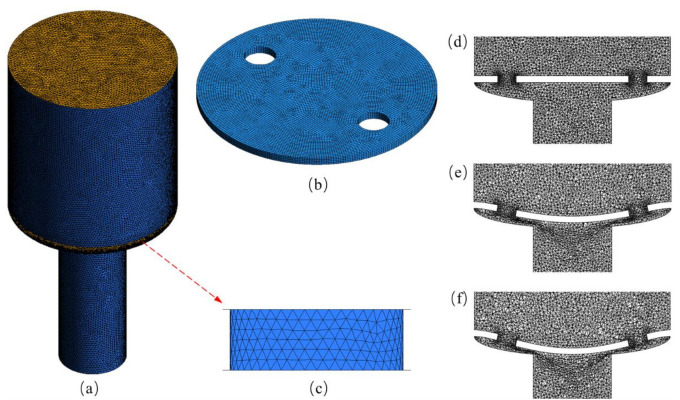
(**a**) Fluid domain mesh, (**b**) solid domain mesh, (**c**) refined mesh of microhole, (**d**) initial state without fluid of MPV, (**e**,**f**) mesh moving process of the fluid domain with the membrane deformation.

In FSI analysis, calculation domain involves fluid domain and solid domain. For the fluid domain, unstructured mesh is adopted, the maximum size is 25 μm, and the microholes are grid-encrypted, as shown in Figure 4a,c. Since the deformation of the membrane changes with the fluid inlet pressure, the moving mesh is adopted. The initial and deformed states of the microvalve model are shown in Figure 4d–f. For the solid domain, a structured mesh is adopted, and the mesh size is 10 μm, as shown in Figure 4b.

For boundary conditions of fluid calculation, the medium in microvalve is water with the density of 998.2 kg/m^3^ and the viscosity of 0.00103 kg/m·s. The inlet of microvalve is defined as the pressure-inlet, and the User Defined Functions (UDF) provided by FLUENT is adopted to specify the inlet pressure increasing linearly as a function of time. The inlet pressure ranges from is 0.5 kPa to 6 kPa, with an increase of 0.5 kPa per second. The outlet pressure is set to be atmospheric pressure. The fluid wall in contact with the membrane is defined as the fluid coupling surface. The ANSYS-FLUENT is used for solving equations based on the SIMPLE algorithm and the second-order upwind scheme. For boundary conditions of solid calculation, elastic membrane material is PDMS. Its properties are shown in Table 1. The membrane surface in contact with the fluid is set as the solid coupling surface, and the remaining surface is set as fixed support.

### 2.4. Numerical Method Validation

**Figure 5:**
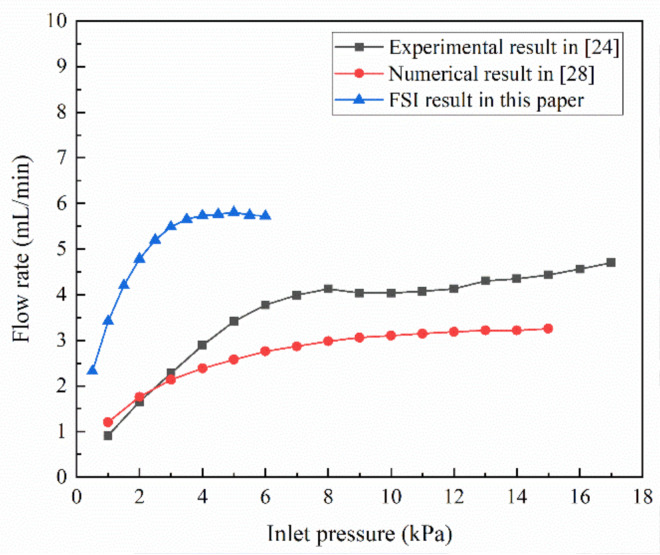
Comparison of experimental results with numerical results varied inlet pressure.

The flow rate in the MPV proposed by Zhang et al. [24] can be estimated as:

(9) $$Q=\frac{P}{R_{f}}=\frac{P+\Delta P}{R_{f}+\Delta R_{f}}$$

where *P* refers to the initial inlet pressure; Δ*P* refers to the pressure gradient; *R_f_* refers to the initial flow resistance of the valve; Δ*R_f_* refers to the resistance increment. It can be found from Equation (9) that, when the inlet pressures increase Δ*P*, the resistance caused by the deformation of the membrane increases Δ*R_f_*. As the inlet pressure is beyond a threshold pressure, pressure of *P* + Δ*P* on the numerator in real time compensates for the resistance of *R_f_* + Δ*R_f_* under the denominator, and the MPV can output a constant flow rate *Q*. This conclusion is proved by the experimental and numerical simulation results of Zhang et al., as shown in Figure 5. However, when the inlet pressure is too high, the deformed membrane is infinitely close to the outlet of the control chamber, until it blocks the outlet of the control chamber. At this time, the flow resistance in the MPV tends to be infinite, and the flow rate is close to zero. The material and structure of the membrane in this paper are different from the literature [24]. In the numerical calculation process, when the inlet pressure reaches about 4 kPa, the flow rate has stabilized, achieving the purpose of a high flow rate control under ultra-low threshold pressure. Meanwhile, when the inlet pressure exceeds 6 kPa, the deformed membrane may completely block the outlet of the control chamber. Therefore, the maximum inlet pressure of the MPV is 6 kPa in this paper.

In this paper, in order to verify the reliability of the two-way FSI analysis, the simulation results for the flow rate in different inlet pressure are compared with Zhang et al. [24,28]. The result is shown in Figure 5. The MPV model with *n* = 4 was used in a FSI analysis in this paper. Comparing the flow rate obtained by the FSI analysis with the experiment by Zhang et al. [24], although the properties of the membrane materials is different, the trend of the flow rate with the inlet pressure is consistent. These verify that the reliability of the FSI analysis results.

## 3. Results and Discussion

### 3.1. Flow Characteristics

**Figure 6:**
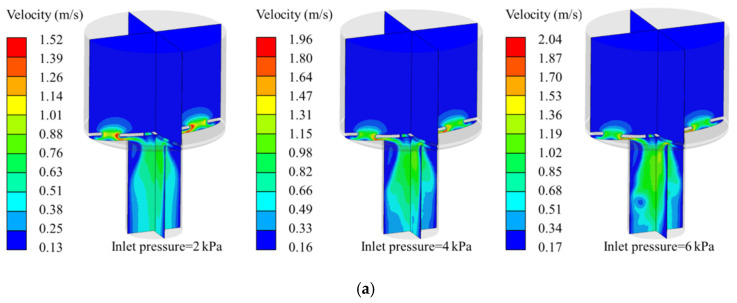

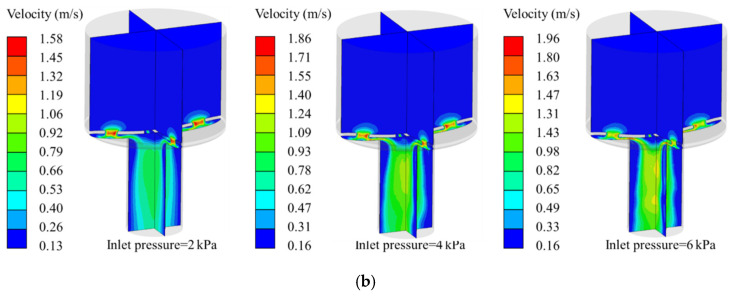
Velocity contours for different inlet pressure in symmetry planes, (**a**) *n* = 2, (**b**) *n* = 4.

In order to study the effect of membrane features on flow characteristics in MPV, different numbers of microholes and pitches of microholes in the members are analyzed by the FSI analysis. The velocity contours for different inlet pressure in symmetry planes are shown in Figure 6. The maximum velocity in the microvalve increases with the increase of inlet pressure. However, it can be found from Figure 6 that the high velocity region first appears at the microholes, and then, the high velocity region is gradually transferred to the outlet of the control chamber as the inlet pressure increases. The deformation of the membrane increases with the increase of the inlet pressure, which makes the membrane gradually approach the inner wall of the flow control chamber. The fluid channel formed by the membrane and the outlet of the control chamber is getting smaller. These indicate that the flow resistance in this region plays a decisive role for the flow rate control of the MPV. Comparing Figure 6a,b, it can be found that the maximum velocity with *n* = 2 has been transferred to the outlet of the control chamber when the inlet pressure reaches 4 kPa, while the maximum velocity with *n* = 4 is still at the microholes under the same conditions. The increase of the number of microholes makes the diameter of the microhole smaller, resulting in a more obvious throttling effect. Therefore, when the inlet pressure is 4 kPa in the microvalve with *n* = 4, the maximum velocity is still in the microholes.

**Figure 7:**
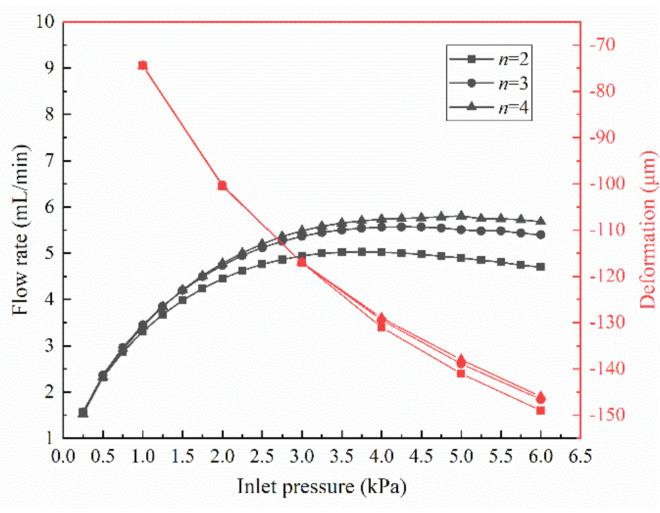
Flow rates and maximum deformation of the MPV with different numbers of microholes under varied inlet pressures (*L* = 1000 μm).

The flow rate of the MPV and the maximum deformation of the membrane are presented in Figure 7. It can be found from Figure 7 that the flow rate rapidly increases at the beginning as the inlet pressure increases. When the inlet pressure exceeds a certain value, the flow rate becomes stable. This shows that the MPV has a pressure range for constant flow regulation, in which it can achieve a good steady flow effect. In order to better analyze the regulation performance of flow rate in the MPV, the constant flow rate, threshold pressure, and pressure range of constant flow rate are shown in Table 2. In this paper, the threshold pressure is defined as the starting pressure when a slope of flow rate nearly achieves zero. Combining Figure 7 and Table 2, it can be found that as the number of microholes on the membrane increase, the constant flow rate increases; correspondingly, the threshold pressure increases. The pressure range of constant flow rate becomes larger. When the number of microholes is increased to 3, the constant flow rate increases by 13%, while, when the number of microholes is increased from 3 to 4, the constant flow rate only increases by 4%. Causing the increase number of the microholes, the diameter of the microhole decrease. Thus, the flow resistance of the microholes increases. However, under the same inlet pressure, the maximum deformation of the membrane decreases with the increase of the number of microholes, which cause the overall flow resistance in the microvalve to decrease instead. This can be confirmed by the curve of deformation with varied inlet pressure in Figure 7. These show that flow regulation in the MPV is very sensitive to the deformation of the membrane.

When the number of microholes on membrane reaches 4 (*n* = 4), the constant flow rate maintained by the microvalve is 5.75 mL/min, and the threshold pressure to achieve the flow rate is 4 kPa. Therefore, compared with the passive microvalve that has been previously reported, the studies of microfluidic passive valve in this work can achieve a higher flow rate at a much lower threshold pressure, as shown in Table 3. On the one hand, this could have attributed to the design of special elliptical control chamber [24], on the other hand it lies in the reasonable design of microholes on membrane and the choice of material properties.

**Figure 8:**
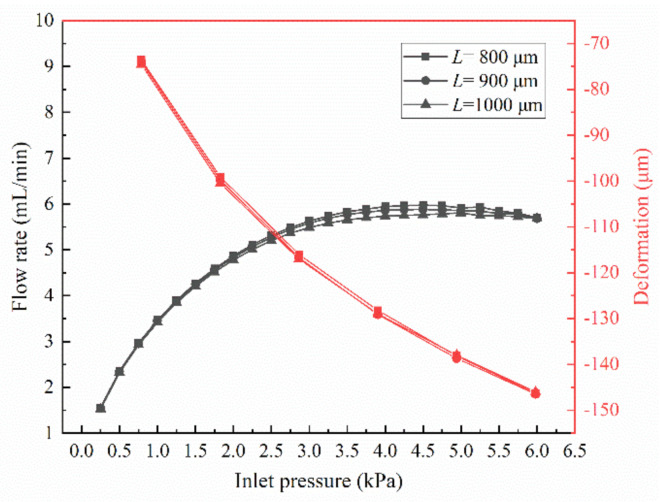
Flow rates and maximum deformation of the MPV with different pitches of microholes under varied inlet pressures (*n* = 4).

Finally, Figure 8 depicts the effect of different pitches of microholes on the flow rate. It can be seen from Figure 8 that the pitch of microholes have a little effect on the flow rate. The maximum fluctuation range of flow rate is about 2.8%. The curves of maximum deformation with the inlet pressure almost coincide. This indicates that, although pitch of microholes is different, the deformation of the membrane is almost the same. At the same time, the flow resistance in the MPV is the same at the same inlet pressure. Therefore, the flow rate in the MPV does not change with the decrease of the pitch of microholes.

### 3.2. Membrane Deformation against Fluid Flow

**Figure 9:**
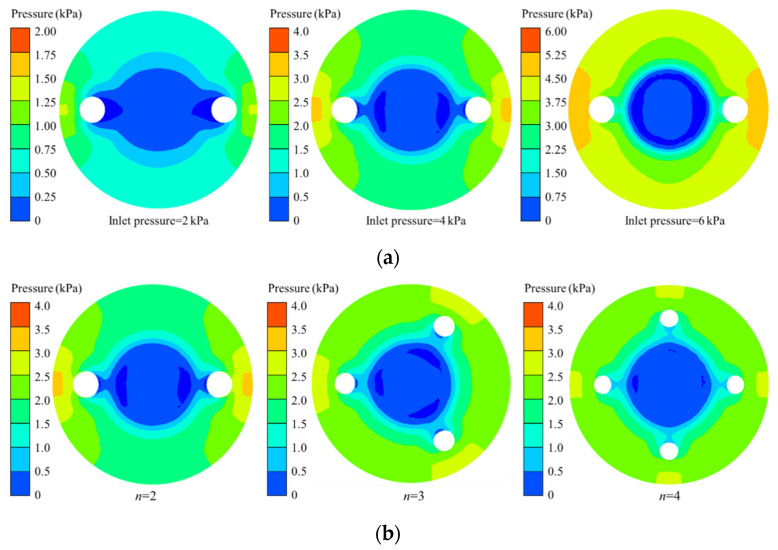
Pressure distribution for lower surface of the membrane in microvalve. (**a**) Different inlet pressure with *n* = 2, (**b**) different number of microholes with inlet pressure of 4 kPa.

In order to evaluate the performance and behavior of the MPV, the pressure distributions at lower surface of membrane are analyzed, as shown in Figure 9. Since the upper surface of the membrane is subjected to uniform inlet pressure, it will not be discussed. It can be found from Figure 9a that as the inlet pressure increases, the low-pressure region on the lower surface of the membrane gradually moves toward the center of the membrane, forming a ring shape. Under the same pressure, as the number of microholes increases, the area of the low-pressure region on the lower surface of the membrane is reduced, as shown in Figure 9a. The pressure on the lower surface of the membrane is evenly distributed in a ring. The region of maximum pressure appears on the periphery of the membrane facing the microholes. Therefore, the number of microholes on the membrane has a non-negligible effect on the fluid flow in the MPV.

**Figure 10:**
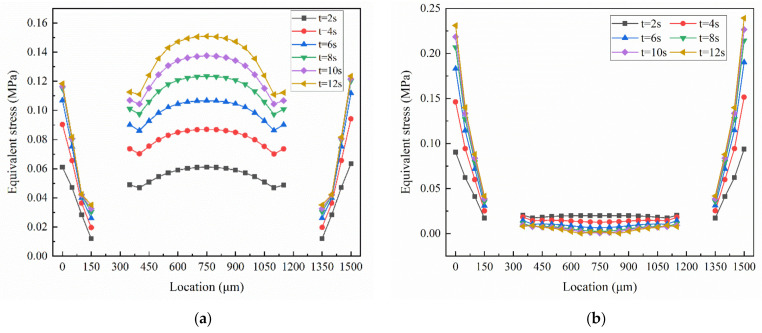
Equivalent stress of the membrane in various time step. (**a**) Lower surface of membrane, (**b**) upper surface of membrane.

In order to further study the force on the membrane, the distribution of equivalent stress on the membrane is analyzed. This is shown in Figure 10. In Figure 10, the place where the curve breaks are the position of the microholes. The equivalent stress distribution on the lower surface of the membrane appears discontinuous at the microholes. The maximum equivalent stress on the lower surface of the membrane appears in the center of the membrane, and it gradually increases with the increase of the inlet pressure. However, as the inlet pressure increases, the trend of the equivalent stress on the lower surface of the membrane to increase is slowing down. It can be found from Figure 10b that the equivalent stress distribution on the upper surface of the microvalve is relatively uniform, due to the smoothness of the inlet fluid flow. The equivalent stress on the edge of the upper surface of the membrane increases with the increase of the inlet pressure, and the increment of the equivalent stress is also decreasing.

**Figure 11:**
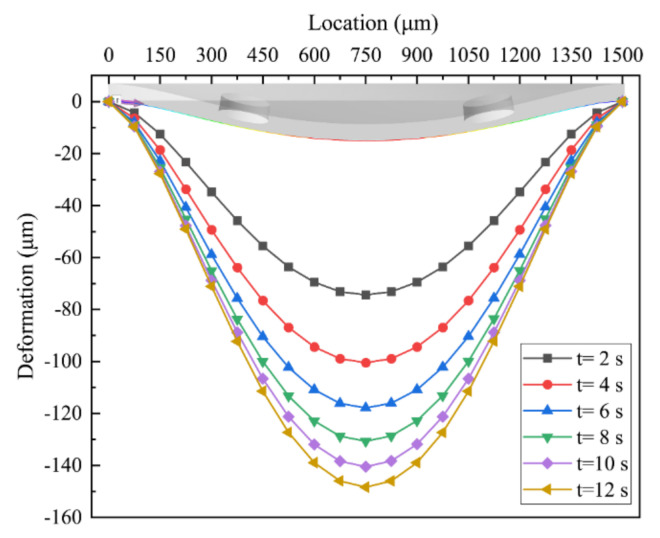
Deformation of the membrane in the path with *n* = 4 and *L* = 1000 μm.

The deformation of the membrane is closely related to the flow regulation performance of the MPV. The deformation of the membrane in the path with *n* = 4 and *L* = 1000 μm is shown in Figure 11. Initially, as the inlet pressure increases, the membrane dramatically deformed, as show in Figure 11. Correspondingly, the slope of the flow rate in the microvalve is higher, as show in Figure 8. When the inlet pressure increases from 1 kPa to 4 kPa, the maximum deformation increased from 75 μm to 138 μm. Subsequently, as the inlet pressure exceeds a certain threshold, the increment of deformation of the membrane slows down and the membrane gradually approaches the inner wall of the control chamber. At this time, the increment of flow resistance in the MPV compensates the increment of inlet pressure in real time, and the corresponding flow slope approaches zero gradually. When the inlet pressure increases from 4 kPa to 6 kPa, the deformation on the membrane increases to 147 μm, and the corresponding deformation increases to 9 μm. Therefore, as the inlet pressure increases, the flow resistance of the microvalve increases rapidly, and the deformation of the membrane gradually becomes stable, i.e., the flow rate across the MPV was regulated to near-constancy.

### 3.3. Effect of Membrane Material Properties on MPV

**Figure 12:**
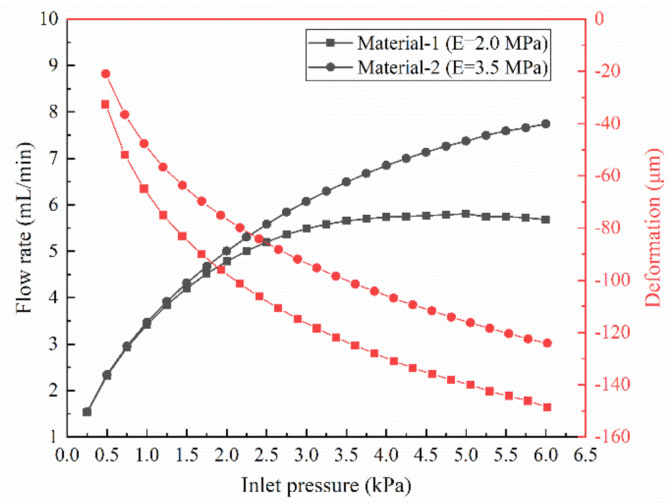
Flow rates and maximum deformation of the membrane with various material properties with *n* = 4 and *L* = 1000 μm.

**Figure 13:**
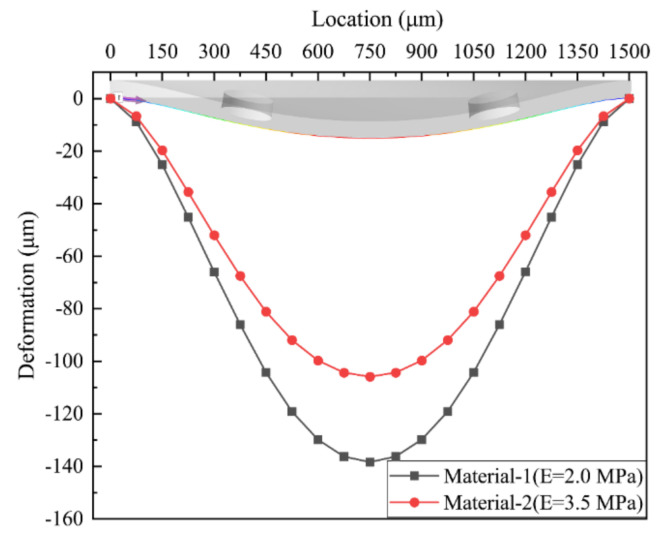
Deformation of the membrane at an inlet pressure of 4 kPa.

In order to study the effect of PDMS material properties on membranes, we selected two PDMSs with different elastic modulus, as shown in Table 1. The maximum deformation of the membrane with varied inlet pressure and the deformation of the membrane at the inlet pressure of 4 kPa are shown in the Figure 12 and Figure 13, respectively. The results clearly indicate that changes in the material properties of the membrane with *n* = 4 cause a decrease in the amount of deformation in both the all stages and the all position of the membrane. The increase in the elastic modulus of the membrane reduces the deformation of the membrane, which leads to a decrease in the flow resistance of the control chamber and a corresponding increase in the flow rate at the same inlet pressure. When the inlet pressure is 4 kPa, the maximum deformation difference of the two different material membranes reaches 23 μm, and the corresponding flow rate difference is 1.1 mL/min. Moreover, the threshold pressure to reach a constant flow value also increases and does not appear within the pressure range studied in this paper. These results also suggest that the flow regulation in the MPV is very sensitive to the deformation of the membrane.

## 4. Conclusions

In this paper, the effect of membrane features on flow characteristics in the MPV and the membrane behavior against the fluid flow were studied using FSI analysis. The microvalve model with different numbers and pitches of microholes were used to performance the flow characteristics of the MPV. The number of microholes on the membrane had non-negligible effects on the fluid flow and flow rate in the MPV. It was found that the constant flow rate maintained by the MPV (*n* = 4) was 5.75 mL/min, and the threshold pressure to achieve the flow rate was 4 kPa. The flow regulation performance of the MPV was better than the passive microvalve that has been previously reported. The change of pitch of microholes in this study had little effect on the flow rate. 

The deformation of the membrane was closely related to the flow regulation performance of the MPV. As the inlet pressure increased, the flow resistance of the microvalve increased dramatically, and the deformation of the membrane gradually became stable, so that the flow rate can be kept constant at a certain range of pressure. When the inlet pressure increased from 1 kPa to 4 kPa, the maximum deformation increased from 75 μm to 138 μm, while as the inlet pressure increased from 4 kPa to 6 kPa, the deformation on the membrane increased to 147 μm, and the corresponding deformation increased to 9 μm.

The effect of membrane material on the flow regulation performance and the deflection of the membrane were studied. Increasing the elastic modulus of the material has an impact on the structural deformation and behavior against fluid flow. Under the same pressure, as the elastic modulus of the membrane increases, the deformation of the membrane became smaller, and the flow rate became larger. When the inlet pressure was 4 kPa, the maximum deformation difference of the two different material membranes reached 23 μm, and the corresponding flow rate difference was 1.1 mL/min.

Finally, under a low threshold pressure, the MPV was able to realize a high flow rate control. This work may provide valuable guidance for the optimization of microfluidic passive valve in a microfluidic system.

## Figures and Tables

**Table 1:** Physical properties of PDMS.

Materials	Density (kg/m^3^)	Elastic Modulus (MPa)	Poisson’s Ratio
Material-1 (PDMS)	960	2	0.49
Material-2 (PDMS)	960	3.5	0.49

**Table 2:** Comparison of regulation performance of flow rate with varying numbers of microholes.

Number of Microholes	Constant Flow Rate (mL/min)	Threshold Pressure (kPa)	Pressure Range of Constant Flow Rate (kPa)
2	5	3.25	(3.25, 4.25)
3	5.54	3.75	(3.75, 5)
4	5.75	4	(4, 6)

**Table 3:** Comparison of the different types of passive flow control valves.

Type	Constant Flow Rate (mL/min)	Threshold Pressure (kPa)
Push-up valve [19]	0.033	103
Parallel membrane valve [20]	0.87	15
Low threshold pressure valve [24]	4.03	6
This work (*n* = 4)	5.75	4
